# N6‐Methyladenosine‐Modified circSMAD4 Prevents Lumbar Instability Induced Cartilage Endplate Ossification

**DOI:** 10.1002/advs.202413970

**Published:** 2025-02-12

**Authors:** Hanwen Li, Yingchuang Tang, Sihan Hu, Xingbang Ruan, Junxin Zhang, Yihan Shi, Liang Qiu, Huilin Yang, Kai Zhang, Hao Chen, Kangwu Chen

**Affiliations:** ^1^ Department of Orthopedic Surgery The First Affiliated Hospital of Soochow University Suzhou Jiangsu 215006 P. R. China; ^2^ Institute of Translational Medicine Medical College Yangzhou University Yangzhou Jiangsu 225000 P. R. China

**Keywords:** cartilage endplate ossification, circSAMD4, IGF2BP1, lumbar instability, m6A modification, Yap1

## Abstract

Lumbar instability causes cartilage endplate ossification and intervertebral disc degeneration. In this study, it is determined that circSMAD4, a Yap1‐related circRNA, is stably downregulated under abnormal stress. In vitro, circSMAD4 knockdown resulted in Yap1 mRNA degradation, whereas circSMAD4 overexpression increased Yap1 mRNA expression and nuclear translocation. Hence, the stabilization of circSMAD4 is essential for maintaining the homeostasis of endplate cartilage under abnormal stress. Furthermore, transcriptome sequencing and mass spectrometry analysis revealed that METTL14‐mediated N^6^‐methyladenosine (m^6^A) modification can stabilize circSMAD4 expression. Moreover, circSMAD4 is shown to regulate Yap1 mRNA through the m6A reader IGF2BP1. The IGF2BP1 functions to translocate Yap1 mRNA into the nucleus, which protects endplate chondrocytes from degeneration. Finally, local injection of an AAV5‐containing circSMAD4 overexpression plasmid successfully rescued LSI‐induced cartilage endplate degeneration, which wasn't observed in Yap1 knockout mice. These findings suggest that m6A‐modified circSMAD4 can stabilize Yap1 mRNA expression and translocation, thus preventing degeneration of the cartilage endplate under abnormal stress. Hence, circSMAD4 may become a potential therapeutic tool for managing instability‐induced intervertebral disc degeneration.

## Graphical abstract

Lumbar instability mediates cartilage endplate degeneration with YAP translocation and stability. Here, it is determined that circSMAD4, a Yap1‐related circRNA, is shown to combine and regulate Yap1 mRNA through the formation of the ternary complexes with the m6A reader IGF2BP1. The ternary complexes function to translocate Yap1 mRNA into the nucleus and protect the lumbar cartilage endplate from degeneration.

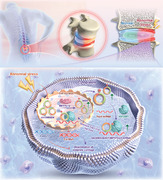

## 1 Introduction

Lumbar degeneration is a global health problem with a high incidence and high cost of treatment.^[^ ^1^ ^]^ Clinically, patients with lumbar degeneration have radiographic changes in the cartilage endplate, namely, Modic changes.^[^ ^2^ ^]^ The intervertebral disc is devoid of vascular and neural tissue, and the endplate is responsible for supplying nutrients.^[^ ^3^, ^4^ ^]^ Histologically, the degenerative cartilage endplate undergoes remodeling, which is also one of the main causes of low back pain (LBP).^[^ ^5^ ^]^ Studies have shown that the elements comprising the lumbar spine are prone to different stressors, which contribute to LBP.^[^ ^6^ ^]^ The mechanical stress of the spine is mainly concentrated in the cartilage endplate (CEP).^[^ ^7^ ^]^ Our previous studies revealed that abnormal stress activates the Hippo signaling pathway and phosphorylates Yap1, resulting in CEP remodeling and intervertebral degeneration.^[^ ^8^ ^]^However, the simultaneous decrease in Yap1 mRNA and protein expression under abnormal stress conditions both in vitro and in vivo may be due to another regulatory mechanism. We speculated that abnormal stress may directly regulate the posttranscriptional processing of Yap1 mRNA.

CircRNAs are a newly emerging class of noncoding RNAs (ncRNAs) that regulate target gene expression directly or indirectly.^[^ ^9^, ^10^ ^]^ In the skeletal system, circRNAs are associated with a variety of biological processes.^[^ ^11^ ^]^ For example, Zhang et al. reported that circVgll3 directly sequesters miR‐326‐5p and promotes Itga5 translation in adipose‐derived mesenchymal stem cells, which may be an effective potential therapeutic target for bone regenerative medicine.^[^ ^12^ ^]^ Cheng et al. demonstrated that circVMA21 acted as a sponge of miR‐200c and indirectly through targeting X‐linked inhibitors of apoptosis in nucleus pulposus cells, which provides a potentially effective therapeutic strategy for intervertebral disc degeneration (IVDD).^[^ ^13^ ^]^ These studies suggest that circRNAs regulate the expression of target mRNAs and proteins to regulate skeletal and chondral disorders. Therefore, in this study, we screened Hippo‐related circRNAs to explore the regulatory effect of abnormal stress on Yap1 mRNA. However, the regulatory mechanism of abnormal stress on circRNAs remains to be studied.

Numerous studies have shown a link between biomechanics and epigenetics, directing cell behavior and fate through the regulation of DNA methylation, histone modifications, and noncoding RNA modifications.^[^ ^14^ ^]^ Moreover, epigenetic alterations play crucial roles in age‐related diseases and conditions, including degenerative spinal diseases.^[^ ^15^ ^]^ N^6^‐methyladenosine (m^6^A) modification, one of the main epigenetic modifications, is the most abundant modification known in eukaryotic mRNAs and noncoding RNAs (ncRNAs)^[^ ^16^ ^]^ and has been confirmed to affect the stability, transport, and transcription of mRNAs.^[^ ^17^ ^]^ M6A modification plays a significant role in the regulation of circRNAs.^[^ ^18^ ^]^ However, whether m6A modification regulates circRNAs and target genes in the process of endplate degeneration is unclear.

In this study, a high‐throughput circRNA sequence with abnormal stress‐stimulated endplate chondrocytes was generated, and circSMAD4, which was significantly downregulated in response to abnormal stress stimulation, was screened. CircSMAD4 regulates the homeostasis of cartilage endplate chondrocytes (CEPCs) by stabilizing Yap1 mRNA. In the cytoplasm, circSMAD4 and Yap1 mRNAs combined to the m6A reader IGF2BP1, which promoted Yap1 mRNA stability and transcription into the nucleus of CEPCs. Our findings suggest that circSMAD4 is a potential therapeutic target for the treatment of lumbar degeneration.

## 2 Results

### 2.1 Profiling of Dysregulated circRNAs in Cartilage Endplate Chondrocytes under Abnormal Stress

**Figure 1:**
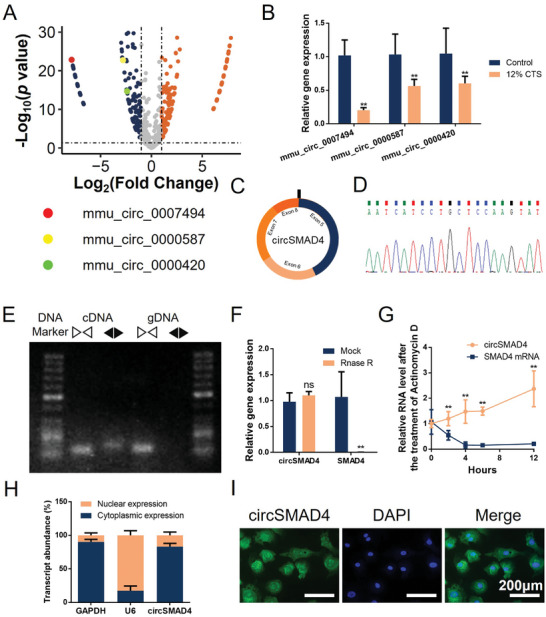
Screening and characterization of circSMAD4. A) Volcano map of dysregulated circRNAs in cartilage endplate chondrocytes (CEPCs) under abnormal stress. B) RT‒qPCR of downregulated circRNAs related to the Hippo pathway. C) Schematic diagram showing the circularization of circSMAD4. D) Sanger sequencing of circSMAD4. E) PCR products with divergent and convergent primers for circSMAD4. cDNA, complementary DNA. gDNA, genomic DNA. F) RT‒qPCR of circSMAD4 and SMAD4 mRNA in CEPCs after treatment with RNase R. G) RT‒qPCR of circSMAD4 and SMAD4 mRNAs after actinomycin D treatment in CEPCs. H) Cytoplasmic/nuclear RNA fractionation of GAPDH, U6, and circSMAD4. GAPDH and U6 were used as positive controls in the cytoplasm and nucleus, respectively. I.RNA fluorescence in situ hybridization (FISH) for circSMAD4. Nuclei were stained with DAPI. ^*^ *p* < 0.05; ^**^ *p* < 0.01.

Based on the sequencing conditions used in our previous experiments,^[^ ^8^ ^]^ we further performed high‐throughput circRNA sequencing in cartilage endplate chondrocytes (CEPCs) under abnormal stress conditions. A total of 233 upregulated and 192 downregulated circRNAs were identified (**Figure** **1**A). Three circRNAs related to the Hippo pathway were downregulated. RT‒qPCR revealed that mmu_circ_0007494 was the most significantly downregulated circRNA (Figure 1B). Given that mmu_circ_0007494 is derived from exons 5–8 within the SMAD family member 4 (SMAD4) locus, we name it circSMAD4 in the subsequent description (Figure 1C). CircSMAD4 was subsequently validated via Sanger sequencing (Figure 1D). After examination via PCR with divergent primers, the sequenced PCR product corresponded to the 5′ exon 5 to 3′ exon 8 (Figure 1E). Afterward, to verify the ring features of circSMAD4, we used ribonuclease R (RNase R), an enzyme that digests linear transcripts, to treat CEPCs. Compared with the parental gene, circSMAD4 was notably resistant to digestion by RNase R (Figure 1F). In addition, after treatment with the inhibitor of transcription actinomycin D, RT‒qPCR analysis revealed that the degradation rate of circSMAD4 was much slower than that of SMAD4 mRNA (Figure 1G). To ensure the cellular localization of circSMAD4, nuclear/cytoplasmic fractionation of RNA was performed in CEPCs. The results suggested that circSMAD4 was located in both the cytosol and the nucleus, while its cytoplasmic distribution was predominant (Figure 1H). Fluorescence in situ hybridization (FISH) revealed that circSMAD4 was localized mainly in the cytoplasm but was also present at lower levels in the nucleus (Figure 1I). These data collectively revealed that circSMAD4 is a circRNA that is abundantly and stably expressed in CEPCs.

### 2.2 CircSMAD4 Maintains the Homeostasis of the Cartilage Endplate

**Figure 2:**
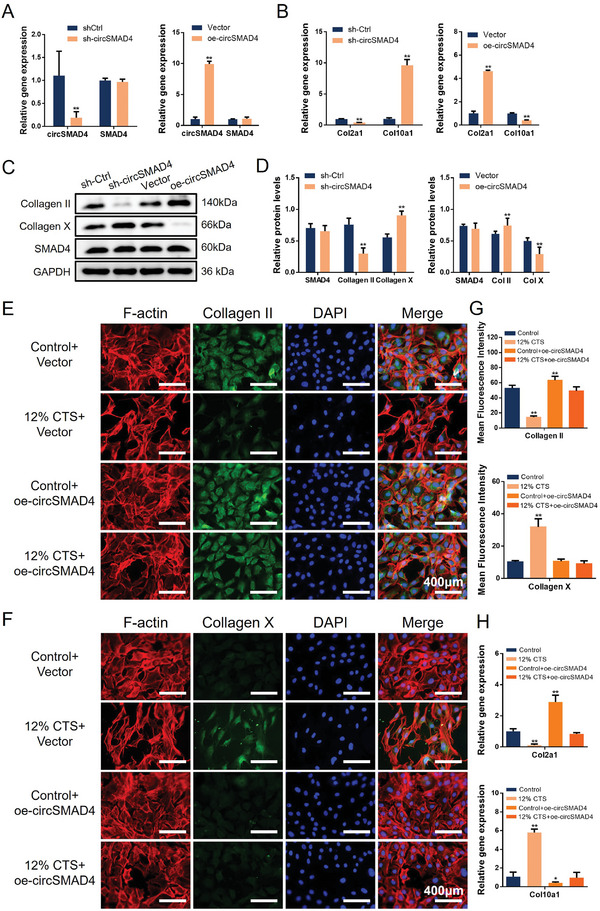
CircSMAD4 is significantly associated with CEPCs homeostasis. A) RT‒qPCR of circSMAD4 and SMAD4 mRNA in CEPCs transfected with shRNA (sh‐circSMAD4) and overexpression plasmids (oe‐circSMAD4). B) RT‒qPCR of Col2a1 and Col10a1 in CEPCs transfected with sh‐circSMAD4 and oe‐circSMAD4. C) WB analysis of the Collagen II, Collagen X, and SMAD4 proteins in CEPCs transfected with sh‐circSMAD4 and oe‐circSMAD4. D) Quantitative analysis of the proteins in (C). E) Representative IF images of Collagen II in circSMAD4‐overexpressing and control cells under abnormal stress. F) Representative IF images of Collagen X in circSMAD4‐overexpressing and control cells under abnormal stress. G) Quantitative analysis of (E) and (F). H) RT‒qPCR of Col2a1 and Col10a1 in circSMAD4‐overexpressing and control plants under abnormal stress. ^*^ *p* < 0.05; ^**^ *p* < 0.01.

To verify the role of circSMAD4 in CEPCs, we constructed shRNA (sh‐circSMAD4) and overexpression plasmids (oe‐circSMAD4) to silence or overexpress circSMAD4 in vitro. The sh‐circSMAD4 efficiently knocked out circSMAD4 without affecting the expression of SMAD4 mRNA, and the downregulation efficiency was greater than 90%. Moreover, overexpression‐circSMAD4 plasmids (oe‐circSMAD4) increased circSMAD4 expression by more than 10‐fold without affecting SMAD4 mRNA expression (**Figure** **2**A). We further evaluated the expression of the cartilage marker Col2a1 and the hypertrophic chondrocyte marker (a type of cartilage degeneration subtype)^[^ ^19^ ^]^ Col10a1. The results revealed that downregulation of circSMAD4 inhibited the expression of Col2a1 and increased the expression of Col10a1, whereas overexpression of circSMAD4 reversed these changes (Figure 2B). In addition, Western blot (WB) analysis revealed that knocking down circSMAD4 directly decreased the expression of collagen II and increased the expression of collagen X in CEPCs without affecting the expression of SMAD4 (Figure 2C,D). Therefore, the lack of circSMAD4 directly leads to CEPCs degeneration in vitro. We further examined the protective effect of circSMAD4 in endplate chondrocytes under abnormal stress. The endplate chondrocytes were first transfected with oe‐circSMAD4 and the control plasmids. Then, a 12% amplitude, 0.5 Hz, and 8 h daily stretch was applied for seven days to simulate the standard stimulus for abnormal stress.^[^ ^8^ ^]^ IF staining revealed that the cells were deformed and agglomerated under abnormal stress stimulation and that the expression of Collagen II was decreased in the 12% CTS group, whereas these phenomena did not occur in the oe‐circSMAD4 group under abnormal stress (Figure 2E,G). In contrast, Collagen X, a degenerative marker in CEPCs, was expressed at significantly higher levels in the 12% CTS group than in the 12% CTS+oe‐circSMAD4 group (Figure 2F,G). The gene expression of Col2a1 and Col10a1 was detected via RT‒qPCR, and the results were similar to those of the IF staining test (Figure 2H). Taken together, these results suggest that circSMAD4 plays an important role in maintaining the homeostasis of cartilage endplate cells.

### 2.3 CircSMAD4 is Exported to the Cytoplasm through METTL14‐Mediated m6A Modification

**Figure 3:**
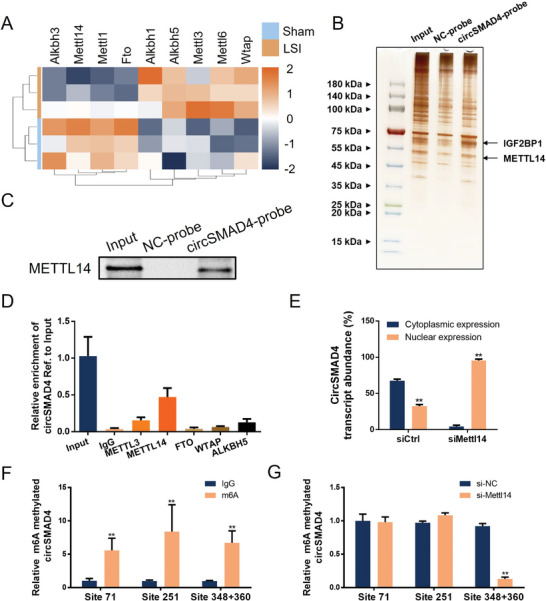
METTL14 promotes the m6A modification and cytoplasmic export of circSMAD4. A) Heatmap of m6A‐related enzymes in the cartilage endplates of the sham and LSI groups. B) RNA pulldown assay and silver staining of CEPCs with biotin‐labeled NC and circSMAD4 probes. C) CircSMAD4 pulldown assay. D) RIP assays with IgG, METTL3, METTL14, FTO, WTAP, and ALKBH5 were performed to evaluate the enrichment of circSMAD4. E) Cytoplasmic/nuclear RNA fractionation experiment of circSMAD4 in siMettl14‐CEPCs and the control. F) Methylated RNA immunoprecipitation (MeRIP) assays were conducted in CEPCs. G) MeRIP assays were conducted in Mettl14‐KD CEPCs. ^*^ *p* < 0.05; ^**^ *p* < 0.01.

N^6^‐methyladenosine (m^6^A), the most abundant epigenetic modification in eukaryotic RNAs, has been shown to play a crucial role in regulating the fate and biological function of circRNAs, thus affecting various physiological and pathological processes.^[^ ^20^ ^]^ To explore the underlying mechanisms involved in the regulation of circSMAD4, transcriptome sequencing of endplate cartilage from the sham or LSI group of mice was performed to examine the enrichment of normal m6A‐related enzymes. The results revealed that the expression of “writers” and “erasers”^[^ ^21^ ^]^ associated with m6A modification was significantly altered under abnormal stress (**Figure** **3**A), suggesting that m6A modification may play a regulatory role in circSMAD4. RNA pull‐down assays and mass spectrometry analysis were used to screen the m6A‐related binding proteins of circSMAD4, revealing that the m6A writer METTL14 and the m6A reader IGF2BP1 were the major binding proteins (Figure 3B and Table S3, Supporting Information). In addition, RNA pull‐down experiments confirmed the binding between METTL14 and circSMAD4 (Figure 3C). RNA‐binding protein immunoprecipitation (RIP) assays revealed that RNA fragments of circSMAD4 were enriched by METTL14 but not METTL3, FTO, WTAP or ALKBH5 (Figure 3D). These results suggest that METTL14 is the main m^6^A regulator of circSMAD4 in CEPCs.

Methyltransferase‐like 14 (METTL14) is the central component of the m6A methylated transferase complex, which is involved in the dynamic reversible process of m6A modification.^[^ ^22^ ^]^ Some reports have demonstrated that m6A modification regulates the biogenesis and nuclear export of circRNAs.^[^ ^23^ ^]^ SiRNAs were used to knock down the expression of Mettl14 in CEPCs. Further cytoplasmic/nuclear RNA fractionation experiments revealed that knockdown of METTL14 significantly increased the proportion of circSMAD4 in the nucleus; that is, circSMAD4 was dependent on the m6A modification of METTL14 for export to the cytoplasm (Figure 3E).

To confirm the regulation of circSMAD4 by m6A modification through METTL14, SRAMP software was used to predict the m^6^A sites of circSMAD4.^[^ ^24^ ^]^ The prediction results revealed 4 “very high” and 3 “high” confidence m6A modification sites on circSMAD4. Methylated RNA immunoprecipitation (MeRIP) assays revealed that anti‐m6A enriched the potential m6A‐modified circSMAD4 “very high” locus (including site 71, site 251 and site 348+360) rather than the IgG locus (Figure 3F). Interestingly, the inhibition of Mettl14 elicited a reduction in m6A enrichment only at site 348+360 (Figure 3G). Taken together, these results confirmed that circSMAD4 relies on METTL14‐mediated m6A modification for export to the cytoplasm.

### 2.4 CircSMAD4 Binds to IGF2BP1 Through m6A Modification

**Figure 4:**
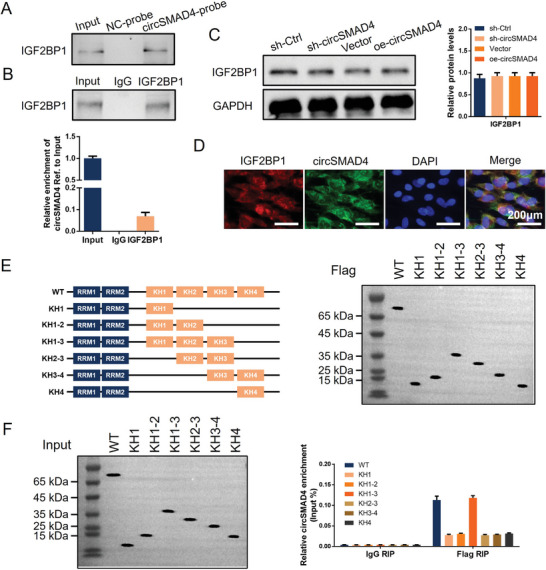
CircSMAD4 interacts with IGF2BP1 via the KH1‐3 domain A) CircSMAD4 pulldown assays. B) RIP assays with anti‐IGF2BP1 and IgG antibodies were performed to evaluate the enrichment of circSMAD4. C) Left, WB analysis of the IGF2BP1 protein in CEPCs transfected with shRNA or the overexpression plasmid. Right, quantitative analysis of proteins on the left. D) IF combined with FISH of IGF2BP1 (IF) and circSMAD4 (FISH) in CEPCs. E) Left, schematic structures showing RNA‐binding domains within the IGF2BP1 protein and a summary of truncated IGF2BP1. Right, WB analysis of CEPCs transfected with plasmids encoding FLAG‐tagged WT or truncated IGF2BP1s. F) RIP assay of the relative enrichment of circSMAD4 associated with truncated IGF2BP1 relative to the input control. ^*^ *p* < 0.05; ^**^ *p* < 0.01.

The dynamic and reversible modulation of m6A modification is determined by m6A writers and erasers; however, m6A must be recognized by various readers to perform downstream biological functions. As m6A readers, the IGF2BP family is known to recognize m6A binding sites on RNA, which then mainly promote RNA stability or influence downstream transcription.^[^ ^25^, ^26^ ^]^ The above mass spectrometry data revealed that IGF2BP1, along with METTL14, binds to circSMAD4. Hence, we further analyzed the interaction and function of IGF2BP1 with circSMAD4 in CEPCs. The interaction between IGF2BP1 and circSMAD4 was verified via RNA pull‐down and RIP assays. The RNA pull‐down data revealed that circSMAD4 was pulled down with abundant IGF2BP1 protein, and RNA immunoprecipitation confirmed the interaction between IGF2BP1 and circSMAD4 (**Figure** **4**A,B). However, the effect of circSMAD4 manipulation on IGF2BP1 expression was tested via Western blotting, which revealed that circSMAD4 does not directly influence IGF2BP1 expression (Figure 4C). Subcellular localization by IF and FISH confirmed the colocalization of circSMAD4 and IGF2BP1 in the cytoplasm of CEPCs (Figure 4D).

IGF2BPs, a conserved family of single‐stranded RNA binding proteins (RBPs), are composed of six canonical RNA binding domains, including two RNA recognition motif (RRM) domains and four K homology (KH) domains.^[^ ^27^ ^]^ To identify the specific domain of IGF2BP1 that combines with circSMAD4, IGF2BP1 mutants with truncations of individual KH domains were constructed (Figure 4E). Studies have confirmed that the KH3–4 di‐domain is indispensable for m6A recognition and binding, whereas KH1–2 might play an accessory role.^[^ ^25^ ^]^ Therefore, the IGF2BP1 mutants were individually applied in the circSMAD4 RIP assays, which were used to confirm whether m6A modification plays a key role in the binding of IGF2BP1 to circSMAD4. The results revealed that the KH1‐3 tridomain of IGF2BP1 is specifically bound to circSMAD4 (Figure 4F). These findings suggest that IGF2BP1 binds to circSMAD4 by recognizing m6A modifications.

### 2.5 CircSMAD4 Promoted the Stability and Expression of Yap1 mRNA

**Figure 5:**
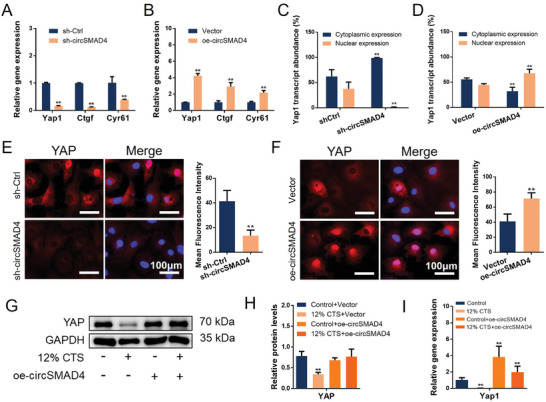
CircSMAD4 promoted the stability and nuclear expression of Yap1 A) RT‒qPCR analysis of Yap1 and downstream genes after circSMAD4 knockdown. B) RT‒qPCR analysis of Yap1 and downstream genes after overexpression of circSMAD4. C) Cytoplasmic/nuclear RNA fractionation of CEPCs with circSMAD4 knocked down Yap1. D) Cytoplasmic/nuclear RNA fractionation of circSMAD4‐overexpressing CEPCs with Yap1. E) Left, IF of YAP in circSMAD4‐knockdown CEPCs. Right, quantitative analysis of the mean fluorescence intensity of YAP. F) Left, IF of YAP in circSMAD4‐overexpressing CEPCs. Right, quantitative analysis of the mean fluorescence intensity of YAP. G) WB analysis of the YAP protein in circSMAD4‐overexpressing and control cells under abnormal stress. H) Quantitative analysis of the proteins in (I). I) RT‒qPCR of Yap1 in circSMAD4‐overexpressing and control cells under abnormal stress. ^*^ *p* < 0.05; ^**^ *p* < 0.01.

In previous experiments, we demonstrated that Hippo pathway‐related circSMAD4 can directly affect the homeostasis of endplate chondrocytes. Our previous study confirmed the role of Yap1 in maintaining homeostasis in endplate chondrocytes under abnormal stress.^[^ ^8^ ^]^ Hence, to determine the relationship between circSMAD4 and Yap1, Yap1 mRNA expression was evaluated in circSMAD4‐silenced or circSMAD4‐overexpressing CEPCs. RT‒qPCR revealed that the knockdown of circSMAD4 reduced the expression of Yap1 and its downstream genes, including Ctgf and Cyr61, whereas the overexpression of circSMAD4 induced the opposite change (**Figure** **5**A,B). These results revealed that the knockdown of circSMAD4 reduced the expression of Yap1 and promoted its degradation.

As a transcriptional regulator, the nuclear translocation status of Yap1 is crucial. Analysis of the nuclear/cytoplasmic fraction revealed that knockdown of circSMAD4 significantly reduced the nuclear ratio of Yap1, whereas overexpression of circSMAD4 promoted its nuclear translocation (Figure 5C,D). In addition, IF revealed that the level of YAP protein expression increased, and YAP translocated into the nucleus when circSMAD4 was overexpressed and decreased when circSMAD4 was silenced (Figure 5E,F). In addition, to verify whether circSMAD4 could maintain Yap1 expression under abnormal stress, CEPCs were mechanically stimulated after transfection with oe‐circSMAD4. WB and RT‒qPCR results revealed that oe‐circSMAD4 could maintain YAP mRNA and protein expression under abnormal stress conditions (Figure 5G–I). Taken together, these findings indicate that circSMAD4 can maintain the stability and expression of Yap1.

### 2.6 CircSMAD4 Stabilizes Yap1 mRNA Expression Through IGF2BP1

**Figure 6:**
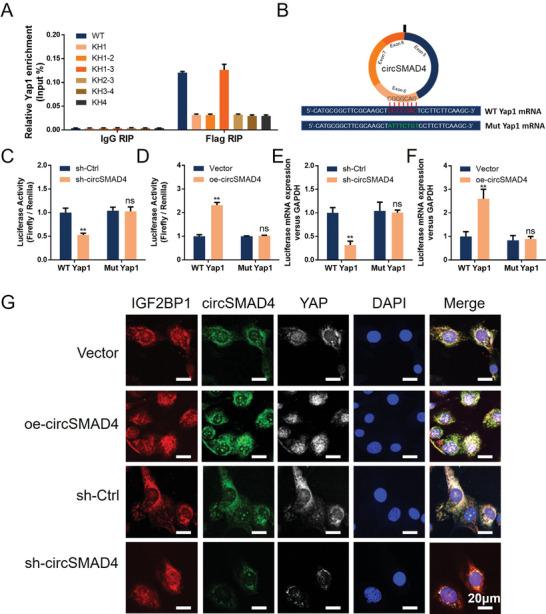
CircSMAD4 stabilizes Yap1 mRNA expression through IGF2BP1 A) RIP assay of the relative enrichment of Yap1 associated with truncated IGF2BP1 relative to the input control. B) Sequence BLAST analysis showing that circSMAD4 directly targets Yap1 mRNA. C) Relative luciferase activity of the luciferase reporter gene with WT‐Yap1 or Mut‐Yap1 in control and circSMAD4‐knockdown HCT116 cells. D) Relative luciferase activity of the luciferase reporter gene with WT‐Yap1 or Mut‐Yap1 in control and circSMAD4‐overexpressing HCT116 cells. E) Luciferase mRNA expression of the luciferase reporter gene with WT‐Yap1 or Mut‐Yap1 in control and circSMAD4‐knockdown HCT116 cells. F) Luciferase mRNA expression of the luciferase reporter gene with WT‐Yap1 or Mut‐Yap1 in control and circSMAD4‐overexpressing HCT116 cells. G) IF‐FISH assay showing the colocalization of circSMAD4, IGF2BP1, and YAP. H) ^*^ *p* < 0.05; ^**^ *p* < 0.01.

As m6A readers, IGF2BPs preferentially recognize m6A‐modified RNAs and promote the stability and translation of thousands of potential mRNA targets in a m6A‐dependent manner.^[^ ^25^ ^]^ Furthermore, IGF2BP1 was reported to play a role in stabilizing mRNA under stress conditions.^[^ ^28^ ^]^ Hence, we speculated that IGF2BP1 functions to transport and regulate Yap1 mRNA in CEPCs. Consistent with the previous experimental approach, the RIP assay revealed that the KH1‐3 tri‐domain of IGF2BP1 is required for its interaction not only with circSMAD4 but also with Yap1 mRNA (Figures 4F, **6**A). To further investigate whether the formation of the circSMAD4/Yap1 complex is vital for Yap1 mRNA expression, BLAST analysis was carried out, which revealed that the CGGCAG within circSMAD4 is a binding site for Yap1 mRNA. Based on this prediction, two luciferase reporters containing wild‐type Yap1 mRNA (WT Yap1) or mutant Yap1 (Mut Yap1) were constructed, in which the GCCCGAC motif corresponding to the binding sequence of circSMAD4 in Yap1 was replaced (Figure 6B). The two plasmids used in the luciferase assay revealed that circSMAD4 knockdown significantly inhibited the luciferase activity of WT Yap1 but not Mut Yap1. Conversely, circSMAD4 overexpression showed the opposite trend (Figure 6C–F). Finally, IF and FISH co‐staining revealed that when circSMAD4 was knocked down, the level of YAP was significantly reduced, whereas circSMAD4 overexpression increased YAP expression and nuclear translocation. However, during this process, IGF2BP1 expression did not change (Figure 6G), indicating that IGF2BP1 acts as a binding protein to maintain the interaction between circSMAD4 and Yap1 mRNA. These data suggest that circSMAD4 mediates the interaction between IGF2BP1 and Yap1 mRNA, which stabilizes Yap1 mRNA and transports it into the nucleus.

### 2.7 CircSMAD4 Prevents Cartilage Endplate Degeneration by Maintaining Yap1 Integrity In Vivo

**Figure 7:**
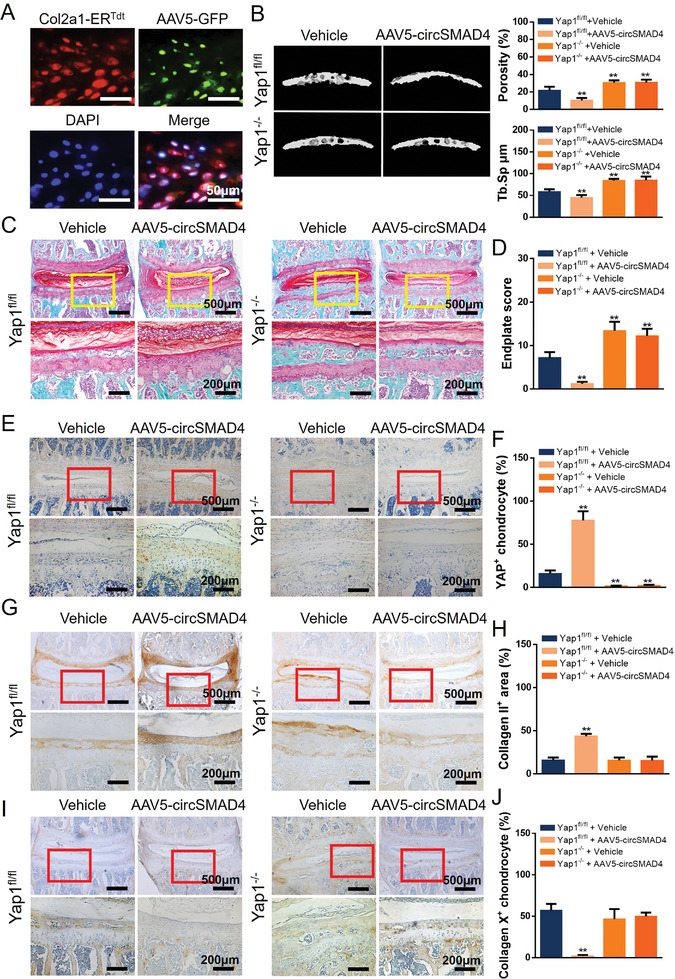
CircSMAD4 protected the cartilage endplate through Yap1 signaling. A) Colocalization analysis of CEPCs (marked with Col2a1‐ER^Tdt^) and AAV5‐GFP. B) Left, representative 3D micro‐CT images of L4/5 mouse caudal endplates (coronal view) at 8 weeks after LSI surgery in different groups. Right, quantitative analysis of the total porosity and trabecular separation (Tb. Sp). C) Representative SO/FG staining images of the CEP. D) Endplate scores of the caudal endplates based on (C). E) IHC analysis of YAP expression in the CEP of different groups of mice at 8 weeks after LSI surgery. F) Statistical analysis of the percentage of YAP‐positive chondrocytes. G) IHC of Collagen II expression in the CEP of mice at 8 weeks after LSI surgery in different groups. H) Statistical analysis of the percentage of the Collagen II area in the CEP. I) IHC of Collagen X expression in the CEP of mice at 8 weeks after LSI surgery in different groups. Statistical analysis of the percentage of collagen X‐positive chondrocytes ^*^ *p* < 0.05; ^**^ *p* < 0.01.

To validate the role of circSMAD4 in the cartilage endplate in vivo, adeno‐associated virus serotype 5 (AAV5) loaded with a circSMAD4 overexpression plasmid (AAV5‐circSMAD4) was used for cartilage endplate injection.^[^ ^5^, ^8^, ^29^ ^]^ The targeting effect of AAV5 was verified via an AAV5‐GFP tracing assay in Col2a1‐CreER::TdTomato (Col2a1‐ER^Tdt^) mice in which CEPCs were labeled.^[^ ^30^, ^31^ ^]^ Colocalization analysis revealed that AAV5 effectively targeted CEPCs (**Figure** **7**A). Genetically manipulated mice with specific Yap1 knockout in endplate cartilage (Col2a1‐ER::Yap1^fl/fl^, Yap1^−/−^) were subsequently used to elucidate the function of circSMAD4.^[^ ^8^ ^]^ AAV5‐circSMAD4 was injected into the endplates of the Yap1^−/−^ and Yap1^fl/fl^ mice after LSI surgery. 3D observations and quantitative analysis via micro‐CT revealed that the porosity and degeneration of the CEP were significantly rescued by AAV5‐circSMAD4 injection in the control group (Yap1^fl/fl^) but not in the Yap1^−/−^ group (Figure 7B). Safranin O and fast green (SO/FG) staining confirmed that AAV5‐circSMAD4 inhibited LSI‐induced endplate cartilage remodeling in the control group but not in the Yap1^−/−^ group (Figure 7C,D). Furthermore, immunohistochemical (IHC) staining was used to verify that YAP expression was significantly reduced after LSI and Yap1 knockout, whereas AAV5‐circSMAD4 injection maintained YAP expression after LSI surgery (Figure 7E,F). Finally, IHC analysis of Collagen II and Collagen X revealed that AAV5‐circSMAD4 maintained CEP homeostasis after LSI surgery in the control group but not in the Yap1^−/−^ group (Figure 7G–J). These results demonstrated that circSMAD4 prevents CEP remodeling or degeneration induced by abnormal stress by maintaining the integrity of YAP.

## 3 Discussion

Disc degeneration‐induced low back pain leads to significant economic and less quantifiable costs worldwide.^[^ ^6^ ^]^ The cartilage endplate (CEP) is the center of stress and the key nutrient supply of the intervertebral disc. CEP calcification is considered one of the initial factors of intervertebral disc degeneration.^[^ ^32^ ^]^ Our previous studies confirmed that the activation of osteoclast differentiation‐related pathways by Yap1 phosphorylation under abnormal stress is the main cause of endplate cartilage remodeling and degeneration.^[^ ^8^ ^]^ However, abnormal stress directly leads to decreases in Yap1 mRNA and protein expression and the degeneration process of chondrocytes, which are not well understood. CircRNAs are formed by back‐splicing of pre‐mRNAs in the nucleus, and they are mainly localized in the cytoplasm.^[^ ^33^ ^]^ They can inhibit miRNA expression, participate in the transcription of target genes, and interact with RNA binding proteins (RBPs).^[^ ^34^ ^]^ In this study, we investigated the role of circRNAs in CEP under abnormal stress. Furthermore, we found that circSMAD4 enhances Yap1 mRNA stability and promotes its nuclear expression. In addition, circRNAs have been confirmed to be associated with a variety of diseases, especially tumors.^[^ ^35^, ^36^ ^]^ Few studies have investigated the role of dysregulated circRNAs in intervertebral discs. Recent studies have focused mostly on the direct control of target gene transcription and expression in the nucleus pulposus.^[^ ^37^, ^38^, ^39^ ^]^ For example, Zhu et al. reported that the level of m6A modification in nucleus pulposus tissue may be associated with the IVDD process.^[^ ^40^ ^]^ Hence, we found that circSMAD4 could directly regulate the homeostasis of endplate chondrocytes to rescue endplate cartilage degeneration caused by abnormal stress, but this process was abolished after Yap1 knockdown. Thus, circSMAD4 can protect CEP from abnormal stress‐induced degeneration through Yap1.

The main epigenetic modifications, including DNA methylation alterations, histone modifications, and dysregulated noncoding RNA modulation, have been implicated in a variety of degenerative diseases.^[^ ^15^ ^]^ N6‐methyladenosine (m6A) is one of the most extensive RNA modifications and can be dynamically regulated by “writers” (methyltransferases), “erasers” (demethylases), and “readers”.^[^ ^41^ ^]^ M6A has been shown to regulate the function and metabolism of circRNAs.^[^ ^20^, ^42^ ^]^ However, little is known about the role of m6A modification in abnormal stress‐induced endplate cartilage degeneration. In this study, we revealed the role of m6A modification on circSMAD4. Mechanistically, circSMAD4 is dependent on METTL14‐mediated m6A modification of the nucleus, which has also been reported in previous studies.^[^ ^43^ ^]^ In the cytoplasm, circSMAD4 is dependent on IGF2BP1, a m6A reader, to enhance the stability of Yap1 mRNA and promote its nuclear expression, thereby maintaining the homeostasis of CEPCs. Recent studies have shown that the knockdown of exportin‐2 increases the nuclear expression of circRNAs and that IGF2BP1 can combine with exportin‐2, assisting in circRNA transport.^[^ ^44^ ^]^ In this study, IGF2BP1 was found to play a trafficking role, but the relationship between IGF2BP1 and exportin‐2 needs to be further confirmed.

As a key factor of the Hippo signaling pathway, YAP has been confirmed to play an important role in cartilage and osteoarthritis.^[^ ^45^ ^]^ YAP activation plays a critical role in maintaining cartilage homeostasis.^[^ ^46^ ^]^ Deng et al. demonstrated that deletion of YAP in chondrocytes promotes cartilage disruption.^[^ ^47^ ^]^ However, these studies did not explain how YAP is degraded during joint degeneration. In this study, we propose a mechanism by which the downregulation of circSMAD4 under abnormal stress leads to decreased stability and nuclear translation of Yap1 mRNA, which may provide a new target for the treatment of degenerative diseases of the articular cartilage.

In summary, we identified circSMAD4 as an important circRNA involved in the maintenance of CEP homeostasis. We further verified that METTL14‐mediated m6A modification promoted the export of circSMAD4 to the cytoplasm. More interestingly, cytosolic circSMAD4 promotes the stability and expression of Yap1 by IGF2BP1, thereby maintaining the homeostasis of endplate cartilage. These findings explain the degradation of YAP in chondrocytes under abnormal stress. More importantly, our study helps elucidate the mechanism of circRNAs in endplate degeneration, which broadens the ideas and options for the treatment of intervertebral disc degeneration diseases.

## 4 Experimental Section

### Isolation of Cartilage Endplate Chondrocytes

Primary CEPCs were procured from the 4–6‐week mouse. In particular, CEP tissues of the mouse lumbar spine were trimmed using sharp scalpel tips. Following 20 min of Trypsin (Gibco) digestion, the plates underwent three washes with sterile PBS. Subsequently, the CEP tissues were transferred to a Collagenase (C6885, Sigma–Aldrich 0.2 mg mL^−1^) solution in a serum‐free medium. After 5–6 h of digestion, the cell suspension was resuspended via centrifugation and cultured in a dish in an F12 medium (10% FBS) with medium changes every 2 days. The obtained cells were characterized by immunofluorescence staining, and the outcomes verified that the extracted cells expressed substantial amounts of Collagen II protein.

### RNA Sequencing

Total RNA was extracted from CEPCs stretched by 12% range, 0.5 Hz, 8 h a day for 7 days and the control group. The RNA sequencing was done by OE Biotech Co. Ltd. (Shanghai, China). Significant differential expression or enrichment was determined utilizing thresholds of *P* < 0.05 and Fold Change > 2 or < 0.5.

### RNase R Treatment

To examine the circular nature and assess the durability of circSMAD4, a total of 2 µg RNA was subjected to treatment with 5 U µg^−1^ RNase R (sourced from Geneseed, Guangzhou, China) for 15 min at 37 °C, followed by RT‐qPCR analysis.

### Actinomycin D Assay

CECs (3 × 10^5^) were placed in a 6‐well plate and incubated overnight, then exposed to 2 µg mL^−1^ actinomycin D (Sigma, USA) for 4, 8, 12, and 24 h. At the specified intervals, cells were collected, and the abundance of circSMAD4 and mRNAs was evaluated utilizing RT‐qPCR.

### RT‐qPCR

Complete RNA from cultivated cells was extracted using FreeZol Reagent (R711, Vazyme, Nanjing, China) and subsequently converted to cDNA utilizing HiScript III All‐in‐one RT SuperMix (R333, Vazyme, Nanjing, China). RT‐qPCR was conducted employing SYBR Green Master mix (Vazyme, Nanjing, China) on a Real‐time PCR Detection System (Bio‐Rad, USA). The results were examined using the ‐ΔCt or 2−ΔΔCt approach.

The primer sequences employed are enumerated in Table S1 (Supporting Information).

### Nuclear and Cytoplasmic Extraction

The separation of cytosolic and nucleic components was performed per the supplier's protocol, employing the materials provided in the Cytosolic and Nuclear RNA Extraction System (21000, Norgen Biotek, Canada).

### RNA FISH

Oligonucleotide‐modified probe sequence for circSMAD4 was procured from Genomeditech (Shanghai, China). Fluorescence in Situ Hybridization Kit for RNA (Beyotime, China) was employed for staining. The probe sequences are depicted below:

circSMAD4: 5′‐FAM‐CATACTTGGAGCAGGATGATTG‐FAM‐3′

### RNA Interference and Transfection

CircSMAD4 overexpression plasmid, shRNAs of circSMAD4, and siRNAs of Mettl14 were synthesized by Genomeditech (Shanghai, China). They were transfected by Lipomaster 3000 (TL301, Vazyme, Nanjing, China). For stable transductions, lentivirus production was performed for oe‐circSMAD4 and shRNAs. The target sequences are depicted in Table S2 (Supporting Information).

### Western Blot

Cellular components and tissue samples were extracted and solubilized using RIPA lysis buffer comprising protease inhibitors. Protein content was ascertained utilizing a BCA Protein Assay Kit (Beyotime, P0012, China). The protein samples underwent SDS‐PAGE separation and were subsequently transferred onto nitrocellulose membranes (0.45 µm, Millipore, USA). Following blocking, the membranes were exposed to primary antibodies overnight at 4 °C, including those against SMAD4 (Proteintech, 10231‐1‐AP), Collagen II (Affinity, AF0135), Collagen X (Abcam, ab182563), METTL14 (Proteintech, 26158‐1‐AP), IGF2BP1 (Proteintech, 22803‐1‐AP), YAP (CST, 14074), and GAPDH (Proteintech, 60004‐1‐IG). After thorough washing, the membranes were incubated with horseradish peroxidase (HRP)‐conjugated secondary antibodies (Goat Anti‐Rabbit IgG, Goat Anti‐Mouse IgG, Beyotime, China) for 1 h at ambient temperature. Subsequently, excess secondary antibodies were eliminated through multiple TBST washes. The resulting antigen‐antibody complexes were visualized utilizing a chemiluminescence detection system. Quantitative analysis of the immunoblots was performed using ImageJ software.

### Silver Staining and Mass Spectrometry Analysis

Silver staining was conducted utilizing the Rapid Silver Stain Kit (Beyotime, China). Mass spectrometry analysis was carried out by Genelily Biotech Co., LTD. Proteome Discoverer software (version 1.4; Thermo Fisher Scientific) was employed for protein identification and quantification. A protein was deemed significant if it exhibited a ratio (circSMAD4/CTRL) exceeding 2 and possessed at least 2 distinct peptides.

### RNA Pull‐Down

Sangon Biotech (Shanghai, China) synthesized the biotinylated circSMAD4 and its complementary sequence. The RNA‐protein complexes were isolated utilizing the Pierce Magnetic RNA‐Protein Pull‐Down Kit (20164, Thermo Fisher Scientific) as per the supplier's protocol. Around 1 × 10^7^ cells were harvested, lysed, and mixed with 100 µL of streptavidin‐coated magnetic particles for 30 min at 4 °C, rotating at 10 rpm min^−1^, along with a biotin‐tagged circSMAD4 probe. The cellular extracts were combined with streptavidin‐coated magnetic particles to capture the biotin‐tagged RNA complexes. The extracted proteins underwent SDS‐PAGE separation followed by silver staining (Fast Silver Stain Kit, Beyotime, China). Subsequently, the distinct silver‐stained protein band in the gel was excised and subjected to trypsin digestion at 37 °C overnight. The resulting peptide samples were desiccated and reconstituted for liquid chromatography‐tandem mass spectrometry (LC‐MS/MS) examination (MaxQuant 1.6.17.0.).

The sequences of the probe for circSMAD4:

5′‐Biotin‐TTGATACTTGGAGCAGGATGATT‐3′

Probe for NC:

5′‐Biotin‐ACGCTGTTGTTCAATTTACTTAC‐3′

### RNA Immunoprecipitation

The RIP experiments were conducted employing the Magna RIP Kit (17‐701, Millipore) per the supplier's protocol. Following the specified protocols, magnetic beads were pre‐coated with antibodies (5 mg). The antibodies utilized in this investigation comprise METTL14 (26158‐1‐AP, Proteintech) and IGF2BP1 (22803‐1‐AP, Proteintech).

### Methylated RNA Immunoprecipitation

The m6A content in circSMAD4 was assessed utilizing the Magna MeRIP m6A Kit (17‐10499) obtained from Millipore. For MeRIP‐qPCR analysis, primers were designed employing SRAMP software (http://www.cuilab.cn/sramp), and their sequences are depicted in Table S1 (Supporting Information).

### Animal Models

The animal procedures in this study were sanctioned by the Ethics Committee of the First Affiliated Hospital of Soochow University (Issue No.2022420). Col2a1‐Cre^ER^ mice were bred with Yap1^flox/flox^ mice. The progeny were then interbred to produce Col2a1‐Cre^ER^:: Yap1^fl/fl^ (conditional ablation of Yap1 in Col2a1 lineage cells, henceforth referred to as Yap1^−/−^ mouse). The Col2a1‐Cre^ER^ mouse strain requires tamoxifen administration as usual. In brief, 3–4w mice received intraperitoneal injections of tamoxifen (T5648, Sigma–Aldrich) at a dosage of 10 mg mL^−1^ for 5 successive days. The mice genotypes were ascertained through PCR analysis of genomic DNA.

LSI surgery was conducted on 8‐week‐old male mice. Before surgical procedures, mice were anesthetized by intraperitoneal injection of pentobarbital sodium. For LSI surgery, the spinous processes, supraspinous and interspinous ligaments of L3‐L5 were completely removed. For sham operations, only L3‐5 paraspinal muscles were dissected. After that, the mice were disinfected with iodine volts again and placed in cages with regular changes of feed, bedding, and water. After 8 weeks, all mice were anesthetized and euthanized, and specimens were obtained.

For AAV administration, AAV5‐circSMAD4 was procured from Genepharma (Shanghai, China). The Yap^−/−^ and Yap^fl/fl^ groups (n = 10 each) were randomly divided into two groups: one group received AAV5‐circSMAD4, and the other group received AAV5 control as Vehicle. For injection, the caudal endplate of L4–L5 was thoroughly exposed using a ventral approach. Subsequently, 1 × 108 AAV particles in a 10 µL volume were injected into the left portion of the caudal endplate of L4–L5 utilizing borosilicate glass capillaries after drilling a hole in the left section of the endplate.

### Micro‐CT

The complete lumbar spine was extracted and examined using Micro‐CT (SkyScan 1176, SkyScan, Aartselaar, Belgium). 3D reconstruction was carried out utilizing system software. Coronal images of the L4–L5 segment were employed to conduct 3D histomorphometric assessments of the caudal endplate. The 3D structural parameters that were evaluated included total porosity and Tb.Sp for the endplates.

### Histomorphological and Immunohistochemical Analysis

The collected samples underwent fixation for 24–48 h, followed by a 2‐week decalcification process. Subsequently, the vertebral column was encased in paraffin or optimal cutting temperature (OCT) medium (Sakura Finetek, Torrance, CA). Coronal slices of the L4–L5 lumbar region, measuring 5 µm in thickness, were prepared for SOFG and immunohistochemical (IHC) staining. Frozen coronal sections of 10 µm thickness were produced for immunofluorescence analysis.

The IHC procedure employed primary antibodies targeting YAP (1:400, 14074, CST), Collagen II (1:400, ab34712, Abcam), and Collagen X (1:400, ab260040, Abcam). A microscope (Zeiss Axiovert 200; Carl Zeiss Inc., Thornwood, NY) was utilized to capture images. Quantitative assessment was performed using ImageJ (NIH) software.

### Statistical Analysis

The evaluations among several groups were conducted utilizing multiple comparisons through one‐way ANOVA. For RT‐qPCR data presented as relative fold changes, Student's t‐test and one‐way ANOVA with Dunnett's test were employed for two‐group comparisons and multi‐group assessment, respectively. Findings are depicted as mean ± s.d. *P* values < 0.05 were deemed significant. All statistical analyses were executed using GraphPad Prism software (Version 7.0).

## Conflict of Interest

The authors declare no conflict of interest.

## Supporting information



Supporting Information

## Data Availability

The data that support the findings of this study are available from the corresponding author upon reasonable request.
