# Supplementary material for: N6‐Methyladenosine‐Modified circSMAD4 Prevents Lumbar Instability Induced Cartilage Endplate Ossification
Source: Adv Sci (Weinh). 2025 Feb 12;12(13):2413970. doi: 10.1002/advs.202413970 (PMC11967797; doi:10.1002/advs.202413970)
Supplement: Supplementary file 1 — Supporting Information [file ADVS-12-2413970-s001.docx]

Table S1. Sequences of the primers used in this work

| Gene symbol | Forward primer | Reverse primer |
| --- | --- | --- |
| mmu_circ_0000587 | AATTAGATTGCGAAACAAGACATAAGCT | TCCATAAGACCCTTCTCCAAGC |
| mmu_circ_0000420 | ACAAGTCTACAAAAGCACATTTCACAT | GAGTTTCTACACCGAGCTCCA |
| mmu_circ_0007494 | CATTTCCAATCATCCTGCTCCAAG | TCCGTTGATGCGCGATTACT |
| SMAD4 | TGCATTCCAGCCTCCCATTT | CCCGAAGGATCCACATAGCC |
| U6 | GCTTCGGCAGCACATATACTAAAAT | CGCTTCACGAATTTGCGTGTCAT |
| GAPDH | TGCACCACCAACTGCTTGC | GGCATGGACTGTAGTCAGAG |
| Col2a1 | GACTGAAGGGACACCGAG | CCAGGGATTCCATTAGAG |
| Col10a1 | GAATTTCTGTGCCAGGAAAACC | TTTTCACCTCTTCTTCCCACTC |
| Mettl14 | CTCCAGGTCGGAGTGTGAAC | AACCGTTTAAACCAGCCCCT |
| Site 71 | TGAAGGACATTCGATTCAAACCATC | GGCGCTGTACGTCTCCGTT |
| Site 251 | GCAGCCATAGTGAAGGACTG | TGATGGTAAGTAGCTGGCTGAG |
| Site 348+360 | CCAGGACAGCAGCAGAATGGA | GGTGTGTATGGTGCAGTCCTA |
| Yap1 | GCTGCAGCAGTTACAGATGG | TGCTCCAGTGTAGGCAACTG |
| Ctgf | GCTGACCTGGAGGAAAAC | ACACCCCGCAGAACTTAG |
| Cyr61 | AGAGGCTTCCTGTCTTTGGC | CCAAGACGTGGTCTGAACGA |

Table S2. Target sequences of the siRNAs, shRNAs and plasmids used in this study

| Gene symbol | Targeted sequence |
| --- | --- |
| siMETTL14#1 | CAGUACCUUUCUUAAGGGA |
| siMETTL14#2 | GCACCUCGGUCAUUUAUAU |
| siMETTL14#3 | GCAUUGGUGCUGUGUUAAAUA |
| sh-Ctrl | TTCTCCGAACGTGTCACGT |
| sh-circSMAD4#1 | TCCAATCATCCTGCTCCAA |
| sh-circSMAD4#2 | ATCCTGCTCCAAGTATGTT |
| sh-circSMAD4#3 | CCTGCTCCAAGTATGTTAG |
| oe-circSMAD4 | CTCCAAGTATGTTAGTGAAGGATGAGTACGTTCACGACTTTGAAGGACAGCCGTCCTTACCCACTGAAGGACATTCGATTCAAACCATCCAACACCCGCCAAGTAATCGCGCATCAACGGAGACGTACAGCGCCCCGGCTCTGTTAGCCCCGGCAGAGTCTAACGCCACCAGCACCACCAACTTCCCCAACATTCCTGTGGCTTCCACAAGTCAGCCGGCCAGTATTCTGGCGGGCAGCCATAGTGAAGGACTGTTGCAGATAGCTTCAGGGCCTCAGCCAGGACAGCAGCAGAATGGATTTACTGCTCAGCCAGCTACTTACCATCATAACAGCACTACCACCTGGACTGGAAGTAGGACTGCACCATACACACCTAATTTGCCTCACCACCAAAACGGCCATCTTCAGCACCACCCGCCTATGCCGCCCCATCCTGGACATTACTGGCCAGTTCACAATGAGCTTGCATTCCAGCCTCCCATTTCCAATCATCCTG |

Table S3. List of top 15 candidates of circSMAD4-interacting proteins that were identified by RNA pull down and mass spectrometry

| **Gene Name** | **Unique peptides** | **Unique sequence coverage [%]** | **Mw(kDa)** | **Accession** |
| --- | --- | --- | --- | --- |
| Actb | 21 | 73.6 | 41.736 | P60710 |
| Tuba1b | 13 | 57.4 | 50.151 | P05213 |
| Pkm | 31 | 58.9 | 57.844 | P52480 |
| Eno1 | 20 | 47.0 | 47.14 | P17182 |
| Igf2bp1 | 25 | 67.6 | 62.279 | O88477 |
| Hspa8 | 28 | 48.6 | 70.87 | P63017 |
| Eef1a1 | 17 | 37.2 | 50.113 | P10126 |
| Tubb4b | 12 | 73.7 | 49.83 | P68372 |
| Akr1b1 | 25 | 21.2 | 35.732 | P45376 |
| Pgk1 | 18 | 45.8 | 44.55 | P09411 |
| Hspa5 | 43 | 53.9 | 72.421 | P20029 |
| Mettl14 | 12 | 51.6 | 52.138 | Q3UIK4 |
| Tkt | 20 | 28.4 | 67.63 | P40142 |
| Hspd1 | 37 | 51.3 | 60.955 | P63038 |
| Tpi1 | 18 | 60.6 | 26.712 | P17751 |
